# Extracellular vesicles from fifth-stage larval *Angiostrongylus cantonensis* upregulate cholesterol biosynthesis and suppress NLRP2-associated inflammatory responses in mouse astrocytes

**DOI:** 10.1128/msystems.01014-24

**Published:** 2024-12-05

**Authors:** Chien-Ju Cheng, Lian-Chen Wang, Lichieh Julie Chu, Kuang-Yao Chen, Ching-Yun Huang, Kuo-Lun Lan, Kuo-Yang Huang

**Affiliations:** 1 Graduate Institute of Pathology and Parasitology, National Defense Medical Center, Taipei City, Taiwan; 2 Department of Parasitology, College of Medicine, Chang Gung University, Taoyuan, Taiwan; 3 Graduate Institute of Biomedical Sciences, Chang Gung University, Taoyuan City, Taiwan; 4 Molecular Medicine Research Center, Chang Gung University, Taoyuan City, Taiwan; 5 Department of Otolaryngology—Head & Neck Surgery, Chang Gung Memorial Hospital, Taoyuan City, Taiwan; 6 Host-Parasite Interactions Laboratory, National Defense Medical Center, Taipei City, Taiwan; 7 Graduate Institute of Medical Sciences, National Defense Medical Center, Taipei City, Taiwan; 8 Department of Pathology, Tri-Service General Hospital, National Defense Medical Center, Taipei City, Taiwan; San Diego State University, San Diego, California, USA

**Keywords:** *Angiostrongylus cantonensis*, extracellular vesicles, host-parasite interaction, cholesterol synthesis, NLRP2 inflammasome

## Abstract

*Angiostrongylus cantonensis* is a zoonotic parasite that causes severe symptoms in humans, including eosinophilic meningitis and eosinophilic meningoencephalitis. Extracellular vesicles (EVs) derived from helminthes have been implicated in regulating host survival and immune response. However, the roles of *A. cantonensis* EVs in modulating parasite pathogenesis and host immune response remain poorly understood. Herein, we characterized EVs derived from *A. cantonensis* fifth-stage larvae (L5) and adult worms. Ultrastructural features showed that EVs from adult worms are smaller in size compared with those from L5. Proteomic analysis identified stage-specific proteins packaged in L5 and adult worm EVs. To investigate the crosstalk between L5 EVs and host cells, RNA sequencing analysis was conducted to identify the differentially expressed genes (DEGs) and enriched biological pathways in mouse astrocytes treated with L5 EVs. GO and KEGG enrichment analysis demonstrated that the pathways related to “cholesterol biosynthesis” are significantly upregulated in L5 EV-treated astrocytes. Based on the transcriptomic data, we observed a downregulated trend of NOD-like receptors (NLRs) protein 2 (NLRP2), a key regulator of brain inflammation, in mouse astrocytes treated with L5 EVs. To validate this result, we utilized ATP to induce the expression of NLRP2 inflammasome-related genes and proteins, as well as the secretion of downstream cytokines. Notably, ATP-induced overexpression of NLRP2 inflammasome-related molecules was significantly reduced in mouse astrocytes upon L5 EV treatment. Collectively, our data suggest that *A. cantonensis* L5 EVs enhance cholesterol synthesis and potentially modulate immune response by reducing NLRP2 inflammasome-related signaling in non-permissive host cells.

**IMPORTANCE:**

*Angiostrongylus cantonensis* is a significant causative agent of eosinophilic meningitis and eosinophilic meningoencephalitis in humans. Helminth-derived extracellular vesicles (EVs) are known to play a crucial role in parasite pathogenesis and host immunomodulation. However, the protein compositions of *A. cantonensis* EVs and their roles in parasite pathogenesis and host immune response remain unclear. Our results demonstrate for the first time the distinct protein compositions in *A. cantonensis* L5 and adult worm EVs. The highly abundant proteins in L5 EVs that have immunomodulatory or pathogenic potential in the host deserve further investigation. Additionally, the uptake of L5 EVs by mouse astrocytes significantly upregulates cholesterol synthesis and suppresses ATP-induced NLRP2 inflammasome-related signaling. This study highlights the immunomodulatory roles of L5 EVs in non-permissive hosts, suggesting their potential as therapeutic targets and vaccine candidates against *A. cantonensis*.

## INTRODUCTION

Angiostrongyliasis is a serious foodborne parasitic disease caused by infection with *Angiostrongylus cantonensis* (1). Rats are the permissive hosts of *A. cantonensis*, where the fifth-stage larvae (L5) migrate from the brain to the lungs and develop into adult worms (2). In non-permissive hosts, such as mice and humans, *A. cantonensis* L5 parasitizes the brain and leads to cerebral angiostrongyliasis, including eosinophilic meningitis and eosinophilic meningoencephalitis (3). Inflammasome activation plays a key role in regulating host inflammation through the release of inflammatory cytokines (4). A recent study reported that *A. cantonensis* infection can activate NLRP1B and NLRC4 inflammasomes, promoting pyroptosis and leading to meningoencephalitis (5). Additionally, CCAAT/enhancer-binding protein (CEBPα) has been shown to activate miR-101b-3p expression, which mediates meningoencephalitis in mice infected with *A. cantonensis* by promoting pyroptosis through the NLRP3 inflammasome (6). Hence, molecules involved in regulating inflammasome-related pathways and pyroptosis may be potential therapeutic targets for treating *A. cantonensis* infection.

Helminths use an array of sophisticated mechanisms to target intracellular machinery in host cells and manipulate host immune responses (7). The immunomodulatory potency of helminths is primarily attributed to their excretory/secretory products (ESPs) (8) that comprise proteins, peptides, organic acids, lipids, carbohydrates, and nucleic acids (9). It has been shown that mouse astrocytes stimulated with the ESPs of *A. cantonensis* L5 induce interleukin 1β (IL-1β) and IL-6 production through the nuclear factor kappa-B (NF-κB) and sonic hedgehog (Shh)-dependent pathways (10). Additionally, *A. cantonensis* L5 ESP treatment elicits autophagy, serving as a protective role in mouse astrocytes in a Shh pathway-dependent manner (11). The Shh pathway is also involved in the activation of endoplasmic reticulum (ER) stress in response to *A. cantonensis* L5 ESP treatment (12). Hence, it is of great importance to identify the components of *A. cantonensis* L5 ESPs and dissect their specific roles in the modulation of host immune responses. A previous proteomic analysis of *A. cantonensis* L5 ESPs identified 254 proteins using two-dimensional gel electrophoresis (2-DE) coupled with matrix-assisted laser desorption ionization time-of-flight (MALDI-TOF), including eight immunoreactivity proteins, such as protein disulfide isomerase, putative aspartic protease, and annexin (13). However, more in-depth proteomic or metabolomic analyses are needed to identify more proteins and metabolites in *A. cantonensis* L5 ESPs, which will provide novel insights into the pathogenesis and therapeutic targets of *A. cantonensis*.

The production of extracellular vesicles (EVs) has been described in nematodes, and their importance during host–parasite interactions has been highlighted (7, 14, 15). For example, exosome-like vesicles secreted from *Brugia malayi* can be internalized by macrophages and activate M1 macrophage polarization (16). Additionally, *Heligmosomoides polygyrus*-derived EVs suppress macrophage activation and the expression of the IL-33 receptor subunit ST2, indicating that EV vaccination generates strong antibody responses and protective immunity against parasite infection (17). *Fasciola gigantica* exosome-like vesicles contain proteins associated with immunomodulation, immune evasion, and virulence, and vaccination with *F. gigantica* exosome-like vesicles protects mice against subsequent infection by *F. gigantica* metacercariae, providing novel strategies for immunotherapy, vaccination, and diagnosis of fascioliasis (18). Additionally, exosome-depleted ESPs of *A. cantonensis* fourth-stage larvae (L4) could induce alternative activation of macrophages via the PI3K-Akt pathway-dependent metabolic reprogramming (19). However, the roles of *A. cantonensis* EVs in the pathogenesis of this parasite and in modulating host immune responses remain poorly understood.

In the present study, we aimed to identify the composition of *A. cantonensis* EVs and elucidate the crosstalk between *A. cantonensis* L5 and host cells via parasite-derived EVs. We purified EVs from both *A. cantonensis* L5 and adult worms, demonstrating that various sizes of EVs are secreted at different developmental stages. Proteomic analysis of *A. cantonensis* L5 and adult worm EVs revealed stage-specific EV cargo. A co-culture system of *A. cantonensis* L5-derived EVs and mouse astrocytes was established, showing that EVs can be internalized by astrocytes and exhibit cytotoxic effects after prolonged incubation. In-depth transcriptomic analysis was conducted to identify differentially expressed genes (DEGs) and distinct biological pathways in mouse astrocytes treated with *A. cantonensis* L5-derived EVs. These results highlighted the potentially important roles of EVs in the pathogenesis of *A. cantonensis* and their impact on modulating host cholesterol biosynthesis and immune responses, which significantly enhances our understanding of the crosstalk between *A. cantonensis* and the non-permissive host via EVs.

## RESULTS

### Isolation and characterization of *A. cantonensis* L5 and adult EVs

**Fig 1:**
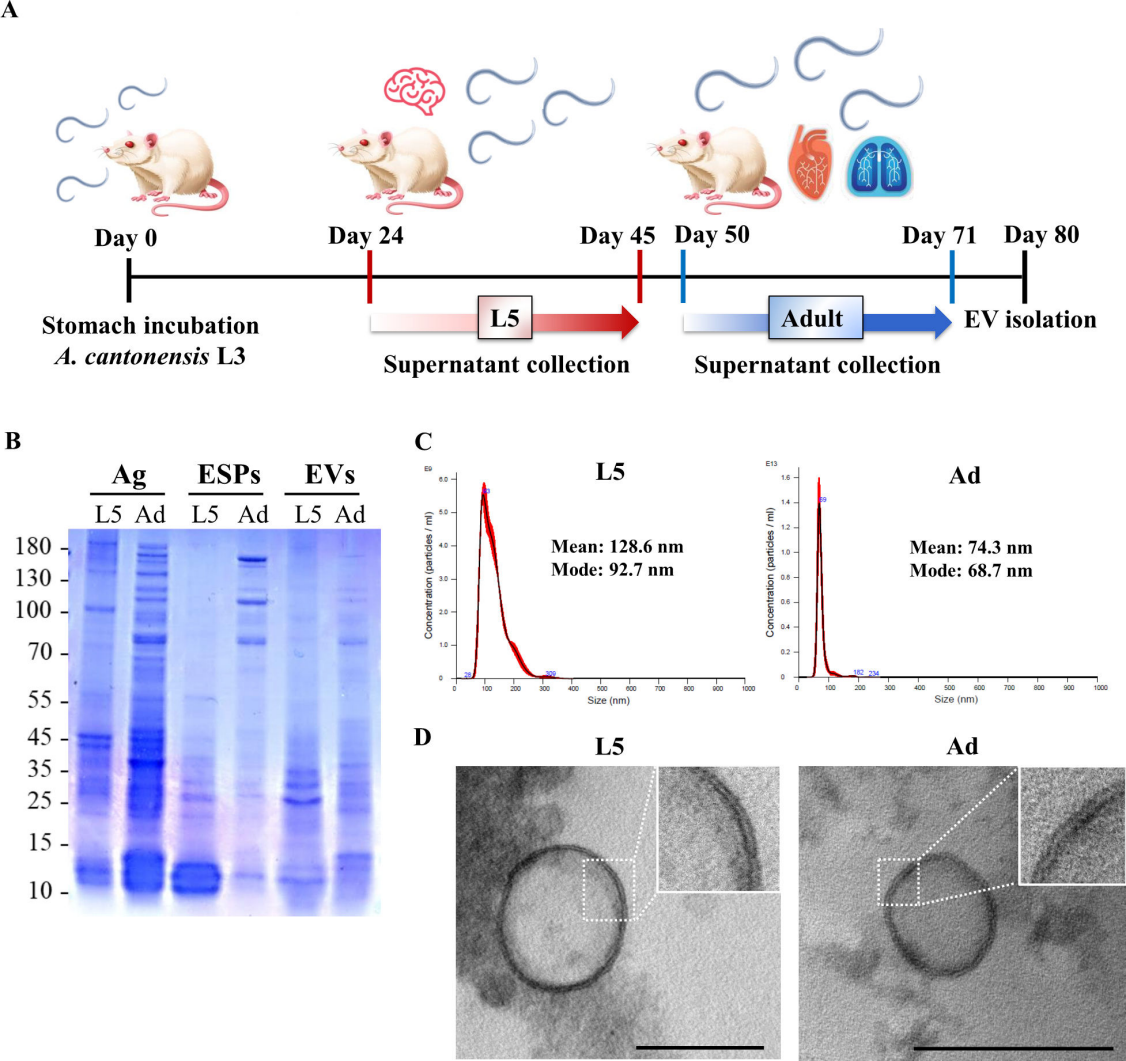
Characterization of EVs isolated from *A. cantonensis* L5 and adult worms. (**A**) Schematic diagram illustrating the collection of EVs derived from *A. cantonensis* L5 (L5) and adult worms (Adult). *A. cantonensis* L3 was used to infect SD rats via stomach incubation. L5 was isolated from the brain tissues of infected rats on days 24–26 post-infection, and adult worms were collected from the heart and lung tissues on days 50–52. The supernatants were collected from the culture medium of L5 and adult worms for EV isolation. (**B**) Coomassie blue staining was used to analyze the protein compositions of antigens (Ag), ESPs, and EVs purified from *A. cantonensis* L5 and adult worms. (**C**) NTA was performed to determine the concentrations and sizes of EVs derived from *A. cantonensis* L5 and adult worms. The mean and mode diameters of EVs derived from L5 and adult worms are shown. (**D**) TEM was utilized to visualize the ultrastructure of EVs secreted from *A. cantonensis* L5 and adult worms. Enlarged images of the highlighted areas show the double-membrane structures of the EVs. Scale bar: 200 nm.

To identify and characterize EVs secreted by *A. cantonensis* L5 and adult worms, L5 was collected from brain tissues on days 24–26, and adult worms were collected from lung and heart tissues on days 50–52 from Sprague Dawley (SD) rats infected with L3 larvae (Fig. 1A). The L5 and adult worms were cultured for 3 weeks, and the culture medium was collected for ESP and EV isolation. The ESPs were purified from both stages as previously described (20). The commercial exosome isolation reagent was used for the rapid and efficient enrichment of EVs, following a previously described method (21). The soluble antigens, ESPs, and the purified fraction of EVs from *A. cantonensis* L5 and adult worms were separated using sodium dodecyl sulfate polyacrylamide gel electrophoresis (SDS-PAGE) (Fig. 1B). As expected, the protein patterns of the purified EVs resembled those of the ESPs. To further analyze the ultrastructure and concentration of EVs, the EV fractions were subjected to electron microscopy (EM) and nanoparticle tracking analysis (NTA), respectively (Fig. 1C). EM analysis revealed the presence of round or cup-shaped vesicles with double membranes in both L5 and adults worms (Fig. 1D), similar to those observed in mammals and other helminths. NTA identified a major population of vesicles in L5 with a concentration of 6 × 10^9^ particles/mL and a mean diameter of 128.6 nm. Notably, EVs from adult worms exhibited a smaller mean diameter of 74.3 nm, suggesting the presence of EVs of varying sizes derived from different stages of *A. cantonensis*.

### Proteomic analysis of *A. cantonensis* L5 and adult EVs

**Fig 2:**
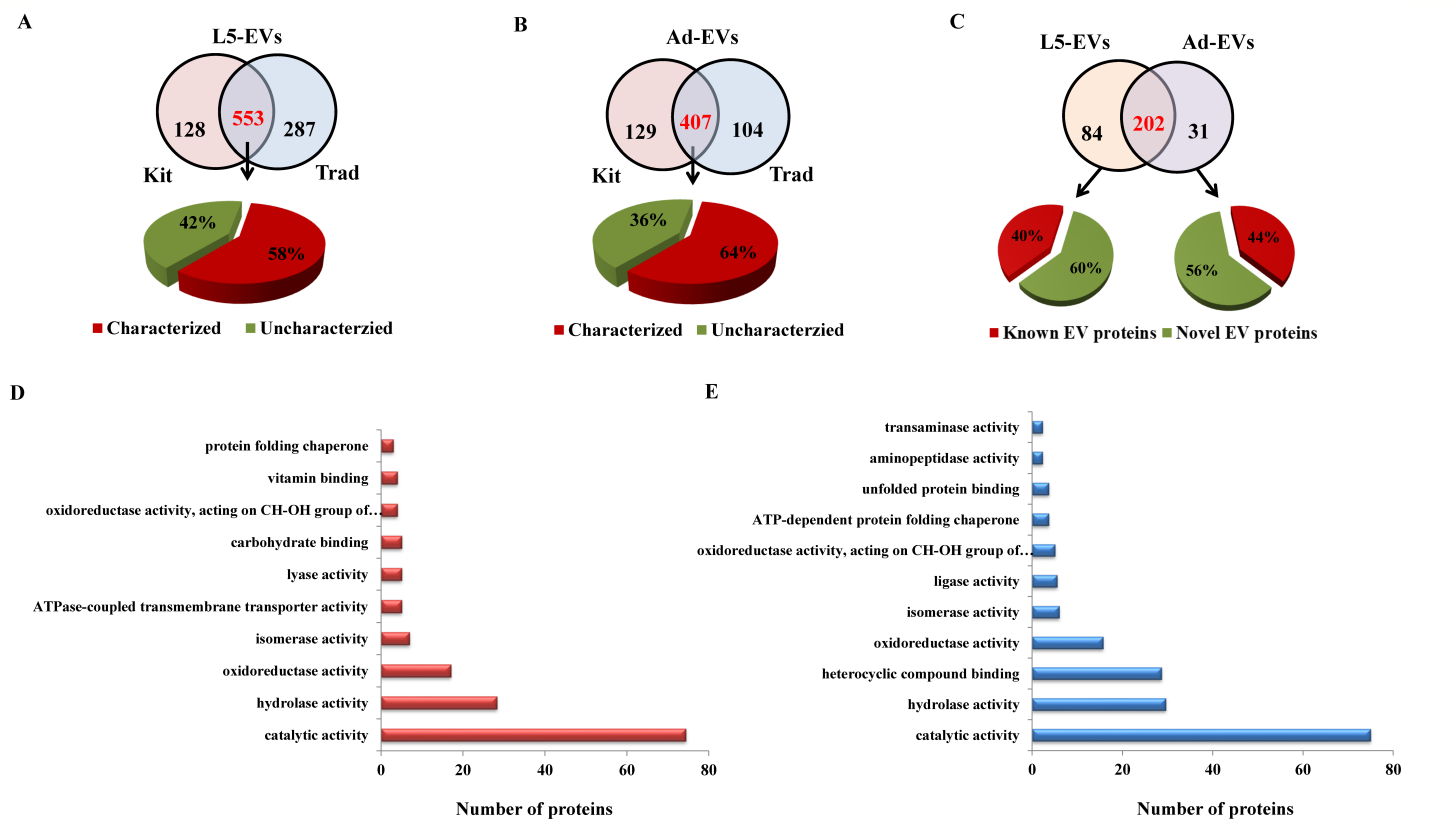
Proteomic analysis of EVs derived from *A. cantonensis* L5 and adult worms. (**A**) EVs secreted from *A. cantonensis* L5 and (**B**) adult worms were purified using differential centrifugation (a traditional method; Trad) and a commercial kit (Kit). The number of identified proteins using the two different isolation methods is shown. The shared proteins were mapped to the *A. cantonensis* database, and the percentages of proteins with and without functional annotations (characterized and uncharacterized, respectively) are presented. (**C**) The percentages of identified EV proteins from *A. cantonensis* L5 and adult worms that match previously known EV proteins (Known EV proteins) are shown. Identified EV proteins from this study that do not match previously known EV proteins are classified as novel EV proteins. (**D**) GO enrichment analysis of EV proteins identified from L5 and (**E**) adult worms. The “molecular function” of EV proteins from L5 and adult worms was analyzed.

To analyze the protein composition of *A. cantonensis* L5 and adult EVs, we employed 1D-LC-MS/MS with an LTQ-Orbitrap mass analyzer. Two isolation methods, including differential centrifugation and a commercial kit, were used to purify the EVs. A total of 553 and 407 common proteins, shared by different isolation methods, were identified from *A. cantonensis* L5 and adult EVs, respectively (Fig. 2A and B). By mapping to the *A. cantonensis* database, 321 and 262 proteins were further characterized (Tables S1 and S2). A total of 202 characterized EV proteins were shared between *A. cantonensis* L5 and adult EVs, while 84 and 31 proteins were unquietly packaged in L5-EVs and adult-EVs, respectively (Fig. 2C). The most abundant proteins in *A. cantonensis* L5 EVs were putative heat shock protein, myosin tail 1 domain-containing protein, annexin, and galectin (Table 1). On the other hand, putative heat shock protein, HATPase_c domain-containing protein, 2-phospho-D-glycerate hydro-lyase, and catalase were highly packaged in adult worm EVs (Table 2). Among these characterized EV proteins shared between L5 and adult worm, approximately 45% were matched to previously identified EV proteins (Table S3), with heat shock proteins, glyceraldehyde-3-phosphate dehydrogenase (GAPDH), and annexin typically found in helminth EVs (18).

**TABLE 1:** Top 20 most abundant proteins in the *A. cantonensis* L5-EV proteome

Accession	Description	Score L5-kit	Score L5-trad	# PSM L5-Kit	# PSM L5-trad
A0A0K0D9H0	Putative heat shock protein	14,648.2	4,411.35	426	118
A0A158P6C3	Myosin_tail_1 domain-containing protein	7,957.23	497.725	264	16
A0A158P8B1	Annexin	8,766.33	8,669.95	262	267
A0A158PB27	Galectin	4,117.49	4,674.38	251	297
A0A0K0D370	Protein disulfide-isomerase	6,291.35	5,777.57	223	196
A0A0K0CUV9	2-Phospho-D-glycerate hydro-lyase	5,755.62	6,059.02	153	165
A0A0K0CSW7	Myosin motor domain-containing protein	4,381.13	1,552.32	152	48
A0A158P7E8	Gal_mutarotas_2 domain-containing protein	3,292.59	4,069.78	129	150
A0A158PBI2	Galectin	2,412.97	2,054.94	112	94
A0A0K0CVJ3	HATPase_c domain-containing protein	3,461.82	3,495.09	106	119
A0A158P9T3	Moesin/ezrin/radixin homolog 1	2,585.19	8,514.43	100	254
A0A0K0DAR9	Alpha-1,4 glucan phosphorylase	2,676.01	2,611.16	93	95
A0A0K0DC78	Rab GDP dissociation inhibitor	2,390.55	2,161.03	92	86
A0A0K0CTU4	Catalase	1,957.85	1,045.31	90	42
A0A0K0CZI7	HATPase_c domain-containing protein	2,869.54	2,838.31	89	94
A0A158P932	Protein disulfide-isomerase	2,375.66	1,880.7	89	77
A0A158PAH5	Ig-like domain-containing protein	1,668.52	1,255.71	84	67
A0A0K0DGX0	Fructose-bisphosphate aldolase	2,626.31	2,927.26	83	98
A0A0K0D232	Protein disulfide-isomerase	3,049.79	1,094.9	74	20
A0A0K0CZV4	Tr-type G domain-containing protein	1,542.32	1,560.36	69	74

**TABLE 2:** Top 20 most abundant proteins in the *A. cantonensis* adult worm EV proteome

Accession	Description	Score Ad-kit	Score Ad-Trad	# PSM Ad-kit	# PSM Ad-Trad
A0A0K0D9H0	Putative heat shock protein	11,649.80	8,975.03	349	271
A0A0K0CVJ3	HATPase_c domain-containing protein	7,444.68	6,449.41	246	217
A0A0K0CUV9	2-Phospho-D-glycerate hydro-lyase	3,919.99	4,226.00	108	102
A0A0K0CTU4	Catalase	2,675.70	2,936.26	106	128
A0A0K0CZI7	HATPase_c domain-containing protein	3,440.51	2,086.57	103	60
A0A0K0DDS4	Fructose-bisphosphate aldolase	2,658.36	3,096.96	94	117
A0A158P8B1	Annexin	3,112.99	4,183.37	84	121
A0A158P9R5	1,4-Alpha-glucan branching enzyme	1,714.66	1,510.96	81	77
A0A0K0DAR9	Alpha-1,4 glucan phosphorylase	1,923.47	2,138.88	80	83
A0A0K0DGX0	Fructose-bisphosphate aldolase	2,223.89	2,160.88	73	68
A0A0K0DDQ6	Tubulin beta chain	2,172.93	1,693.39	67	50
A0A0K0CZV4	Tr-type G domain-containing protein	1,635.74	493.83	67	32
A0A0K0DC78	Rab GDP dissociation inhibitor	1,795.31	1,874.17	66	76
A0A0K0DMA6	Glutamate dehydrogenase	1,532.57	2,152.12	63	87
A0A0K0CXC1	Vesicle-fusing ATPase	1,505.02	782.89	59	28
A0A0K0D9J3	Aspartyl protease inhibitor	1,593.43	1,807.78	58	70
A0A0K0DJC2	SHSP domain-containing protein	1,397.83	1,146.27	58	55
A0A0K0CXW0	Transket_pyr domain-containing protein	1,525.93	1,158.12	58	49
A0A0K0DK12	Glyceraldehyde-3-phosphate dehydrogenase	1,640.40	1,333.72	58	47
A0A0K0DFM8	UTP–glucose-1-phosphate uridylyltransferase	1,652.42	1,086.21	58	42

We next investigated the biological functions of characterized proteins in L5 and adult worm EVs, which were classified into different functional categories based on the “molecular function” ontology through GO enrichment analysis. Among the identified proteins with functional annotations, the major protein families in L5 and adult worm EVs were found to be involved in “catalytic activity” and “hydrolase activity” (Fig. 2D and E). Interestingly, certain proteins with “ATPase-coupled transmembrane transporter activity,” “lyase activity,” and “carbohydrate binding” were specifically enriched in L5 EVs (Fig. 2D), whereas heterocyclic compound binding, ligase activity, aminopeptidase activity, and transaminase activity were more abundant in adult worm EVs (Fig. 2E). Collectively, the proteomic analysis of *A. cantonensis* L5 and adult worm EVs suggests that the differences in protein cargo likely contribute to specialized biological functions in non-permissive and permissive hosts.

### The effect of *A. cantonensis* L5 EVs on the survival of mouse astrocytes

**Fig 3:**
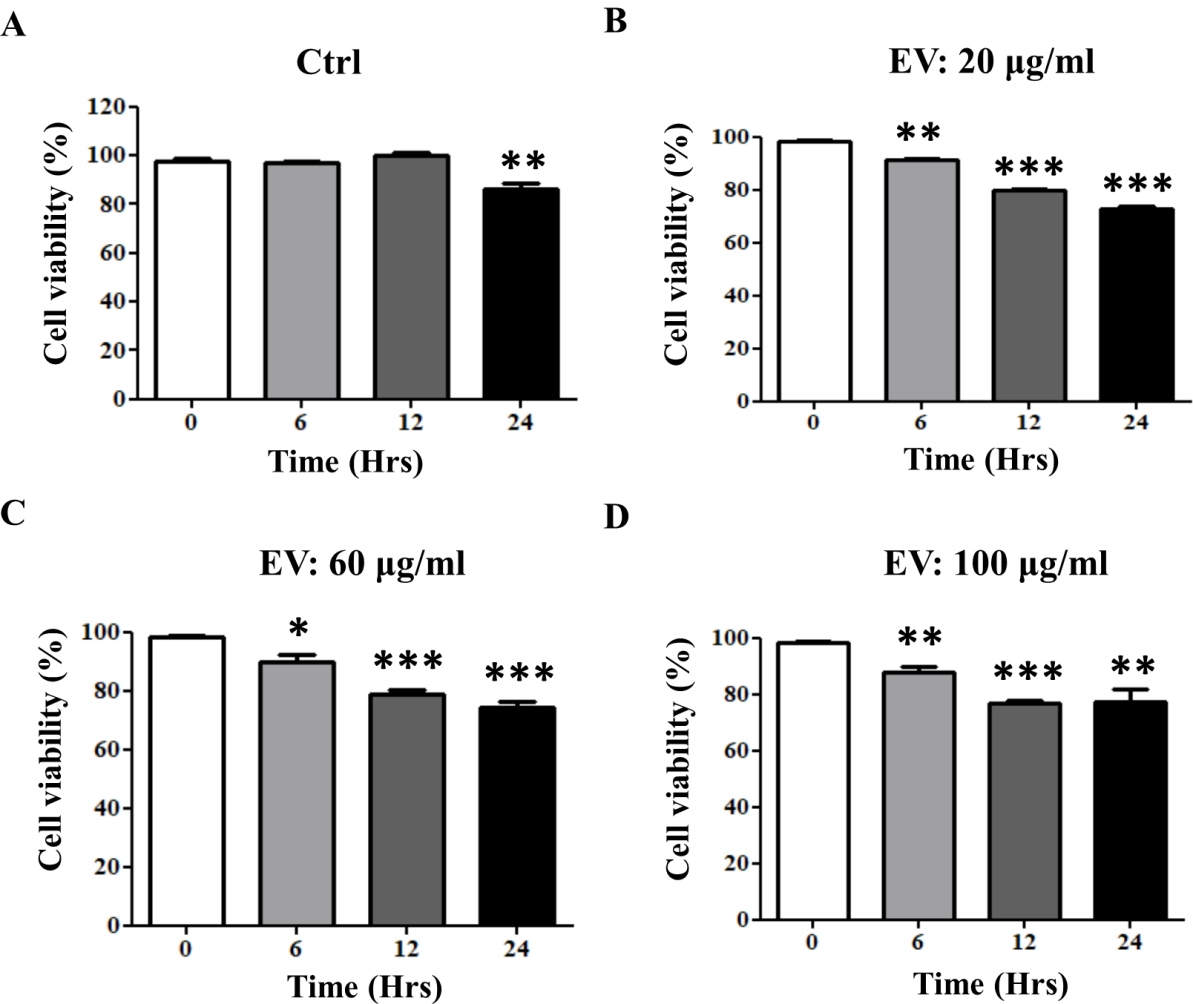
The effects of *A. cantonensis* L5 EVs on the viability of mouse astrocytes. Mouse astrocytes were treated with different concentrations of *A. cantonensis* L5 EVs: (**A**) 0 µg/mL (control; Ctrl), (**B**) 20 µg/mL, (**C**) 60 µg/mL, and (**D**) 100 µg/mL, for various time intervals (6, 12, and 24 h). Cell viability was determined, and the quantitative data are expressed as the means ± SEM from three independent experiments. *P < 0.05, **P < 0.01, ***P < 0.001.

To investigate the effects of *A. cantonensis* L5 EVs on the survival of non-permissive host cells, different concentrations (0, 20, 60, and 100 µg/mL) of EVs were co-cultured with mouse astrocytes for different time intervals (6, 12 and 24 h) (Fig. 3A through D). Mouse astrocytes treated with either low or high concentrations of *A. cantonensis* L5 EVs for 6 h maintained viability above 85% compared with the medium-treated controls. However, the viability of astrocytes remarkably decreased after prolonged incubation with *A. cantonensis* L5 EVs for 12 and 24 h, dropping to 75% and 71%, respectively (*P* < 0.01). These results suggest that *A. cantonensis* L5 EVs have cytotoxic effects on mouse astrocytes after 12 h of treatment.

### *A. cantonensis* L5 EVs are engulfed by mouse astrocytes

**Fig 4:**
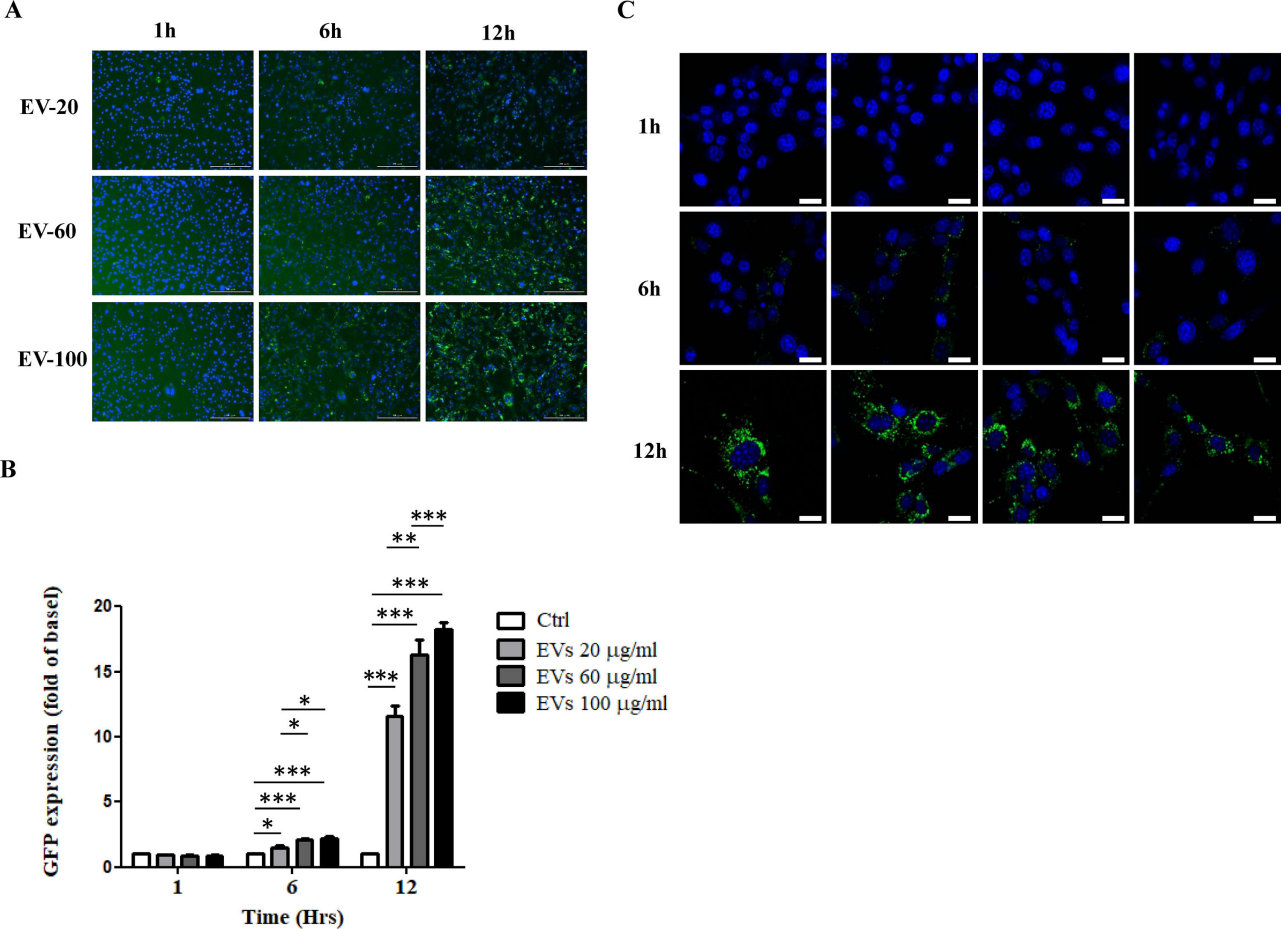
Internalization of *A. cantonensis* L5 EVs by mouse astrocytes. Mouse astrocytes were co-cultured with 20 µg/mL (EV-20), 60 µg/mL (EV-60), and 100 µg/mL (EV-100) of PKH67-labeled EVs from *A. cantonensis* L5 for different time intervals (1, 6, and 12 h). Cellular uptake was visualized using a fluorescence microscope. Scale bar：200 µm. (**B**) Quantitative results of GFP expression in mouse astrocytes treated with PKH67-labeled EVs from *A. cantonensis* L5 under the conditions mentioned in panel **A** compared with the medium-treated control (Ctrl). (**C**) Mouse astrocytes were treated with PKH67-labeled EVs (60 µg/mL) from *A. cantonensis* L5 for 1, 6, and 12 h, followed by visualization under a confocal microscope. Scale bar: 20 µm. *P < 0.05, **P < 0.01, ***P < 0.001.

Pathogen-derived EVs are able to affect the physiological functions of the host by delivering their contents. To examine whether *A. cantonensis* L5 EVs can be internalized by mouse astrocytes, purified EVs were labeled with PKH67 fluorescent dye that incorporates into their lipid-rich membrane. The fluorescent signals of PKH67-labeled EVs were clearly detected in mouse astrocytes after co-incubation for 6 h (Fig. 4A and B). The uptake of EVs by astrocytes was strongly enhanced after prolonged incubation for 24 h. The increased GFP signals in recipient cells indicate a higher level of EV internalization by mouse astrocytes, likely resulting from the elevated EV concentration and extended incubation period. Confocal images confirmed that punctate signals of EVs (60 µg/mL) were observed in the cytoplasm of mouse astrocytes after co-incubation for 12 h (Fig. 4C). These results demonstrate that *A. cantonensis* L5 EVs are significantly internalized by mouse astrocytes after 12 h of co-incubation.

### Transcriptomic analysis of mouse astrocytes treated with *A. cantonensis* L5 EVs identify differentially expressed proteins

**Fig 5:**
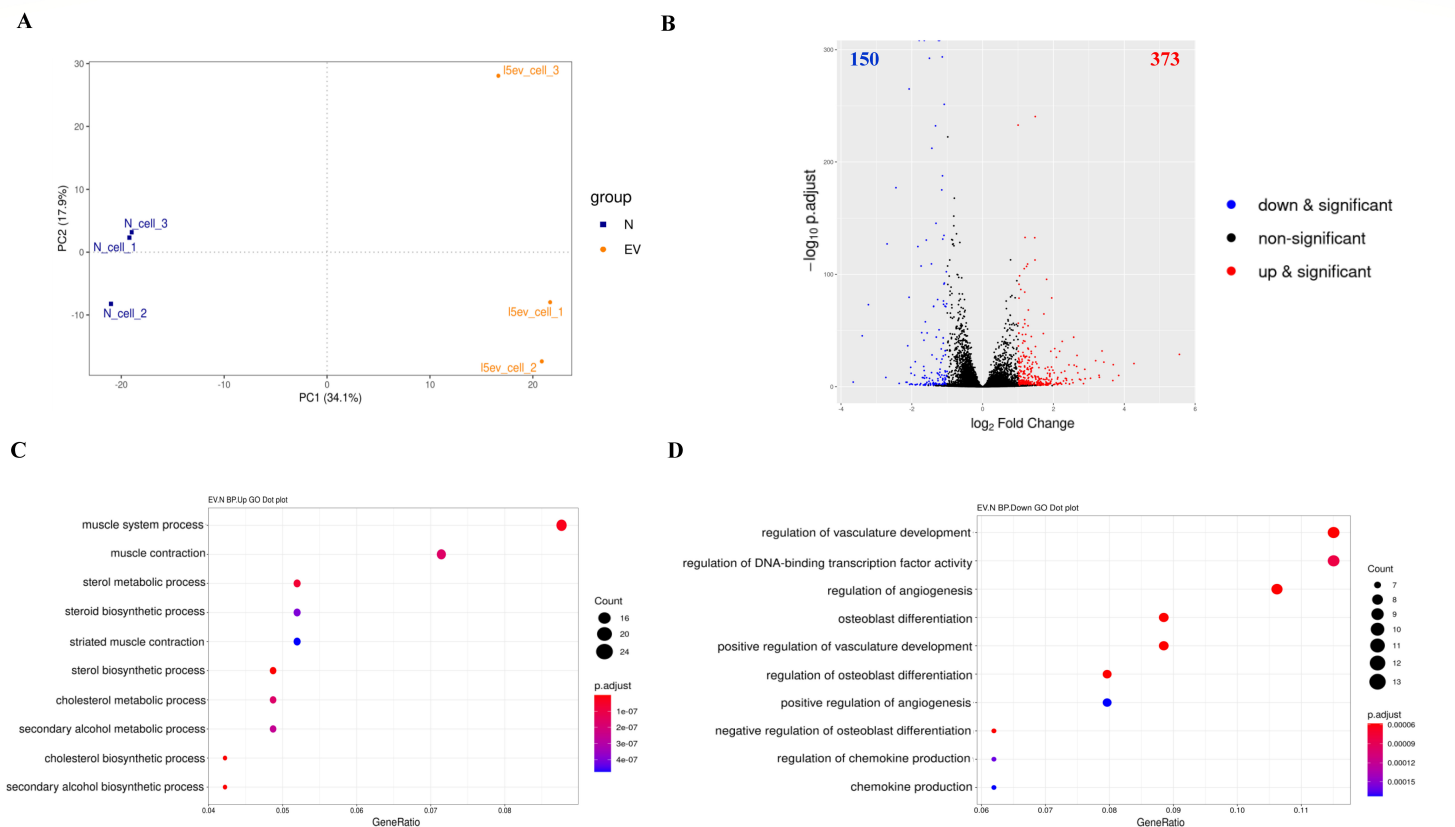
Differential gene expression and GO enrichment analysis of mouse astrocytes treated with *A. cantonensis* L5 EVs. (**A**) PCA of mouse astrocytes treated with *A*. *cantonensis* L5 EVs (EV) compared with the medium-treated control (N). Biological triplicates of mouse astrocytes treated with L5 EVs (60 µg/mL) for 12 h are represented in yellow, while medium-treated controls (N) are shown in blue. (**B**) Volcano plots showing the differentially expressed genes (DEGs) (|log_2_ fold change| ≥ 1) in mouse astrocytes treated with *A. cantonensis* L5 EVs. Blue and red dots represent downregulated and upregulated genes, respectively. The numbers of significantly downregulated and upregulated genes are shown. (**C**) GO enrichment analysis of DEGs revealed upregulated and (**D**) downregulated GO terms from biological processes (BP) in the transcriptome of L5 EV-treated astrocytes compared with the medium-treated control.

As *A. cantonensis* L5 EVs can be taken up by mouse astrocytes, we next assessed the transcriptional changes of astrocytes in response to L5 EVs. We conducted RNA-seq analysis of mouse astrocytes treated with L5 EVs (60 µg/mL) for 12 h compared with the medium-treated control. The mRNA populations derived from biological triplicates were sequenced and mapped to the *Mus musculus* genome. Approximately 354 raw million reads and 53.2 G raw bases were obtained (Table S4). The average percentage of uniquely mapped reads for the 6 RNA samples was 90.38%, generating over 290 million uniquely mapped reads (Table S5). The general expression profile of each sample was determined by principle component analysis (PCA), revealing a clear separation between L5 EVs-treated and non-treated astrocytes (Fig. 5A). We globally analyzed the DEGs in the transcriptome of L5 EVs-treated astrocytes compared to non-treated astrocytes using DESeq2 (*P*-adjust < 0.05, |log_2_ fold change| ≥ 1), identifying 523 DEGs, including 373 upregulated and 150 downregulated genes (Fig. 5B) (Tables S6 and S7). The most upregulated genes in *A. cantonensis* L5 EV-treated astrocytes were the Na^+^/K^+^ transporting ATPase interacting 4 (Table 3), regulator of G-protein signaling 14, and collagen, whereas the most downregulated genes were potassium voltage-gated channel, subfamily F, member 1 (KCNF1), natriuretic peptide type B (BNP), and NLR family pyrin domain containing 2 (NLRP2) (Table 4).

**TABLE 3:** Top 20 upregulated genes in mouse astrocytes treated with *A. cantonensis* L5 EVs

Gene symbol	Description	Log_2_ fold change
Nkain4	Na^+^/K^+^ transporting ATPase interacting 4	5.56
9630013A20Rik	RIKEN cDNA 9630013A20 gene	4.27
Rgs14	Regulator of G-protein signaling 14	3.84
Col9a2	Collagen, type IX, alpha 2	3.69
Mymk	Myomaker, myoblast fusion factor	3.68
Olfr12	Olfactory receptor 12	3.42
Bfsp2	Beaded filament structural protein 2, phakinin	3.37
Hmgcs2	3-Hydroxy-3-methylglutaryl-coenzyme A synthase 2	3.30
Myog	Myogenin (source: MGI; symbol: Acc:MGI:97276)	3.29
Cmtm5	CKLF-like MARVEL transmembrane domain containing 5	3.19
Rab11fip4	RAB11 family interacting protein 4 (class II)	3.05
Lypd6	LY6/PLAUR domain containing 6	2.89
Rgcc	Regulator of cell cycle	2.88
Rcvrn	Recoverin	2.85
Asb2	Ankyrin repeat and SOCS box-containing 2	2.77
Myh3	Myosin, heavy polypeptide 3, skeletal muscle, embryonic	2.74
Gm28653	Predicted gene 28653	2.69
Fbp1	Fructose bisphosphatase 1	2.67
Abca9	ATP-binding cassette, sub-family A (ABC1), member 9	2.65
Slc16a8	Solute carrier family 16 (monocarboxylic acid transporters), member 8	2.57

**TABLE 4:** Top 20 downregulated genes in mouse astrocytes treated with *A. cantonensis* L5 EVs

Gene symbol	Description	Log_2_ fold change
Gm47939	Predicted gene, 47939	−3.66
Kcnf1	Potassium voltage-gated channel, subfamily F, member 1	−3.40
Gm18757	Predicted gene, 18757	−3.23
Gm13522	Predicted gene 13522	−2.73
Nppb	Natriuretic peptide type B	−2.70
Nlrp2	NLR family, pyrin domain containing 2	−2.45
Grem2	Gremlin 2, DAN family BMP antagonist	−2.36
Selp	Selectin, platelet	−2.16
C430002N11Rik	RIKEN cDNA C430002N11 gene	−2.14
Ngef	Neuronal guanine nucleotide exchange factor	−2.12
Siglecg	Sialic acid binding Ig-like lectin G	−2.08
Cyr61	Cysteine-rich protein 61	−2.08
Nobox	NOBOX oogenesis homeobox	−2.06
Nxph3	Neurexophilin 3	−2.03
Plau	Plasminogen activator, urokinase	−2.02
Sprr2b	Small proline-rich protein 2B	−2.02
Gm35940	Predicted gene, 35940	−1.99
Gm12715	Predicted gene 12715	−1.92
Serpinb2	Serine (or cysteine) peptidase inhibitor, clade B, member 2	−1.90
Gm23136	Predicted gene, 23136	−1.89

### Functional enrichment analysis of mouse astrocytes treated with *A. cantonensis* L5 EVs

To investigate the enriched biological processes and molecular functions in astrocytes treated with *A. cantonensis* L5 EVs, we analyzed the DEGs in the transcriptome of L5 EV-treated astrocytes using GO enrichment analysis. Notably, several biological processes related to “steroid and cholesterol biosynthesis” were significantly upregulated in L5 EV-treated mouse astrocytes (Fig. 5C) (Table S8). Additionally, muscle system-related processes, including muscle contraction, muscle cell development, and muscle cell differentiation, which are related to the molecule function of “cytoskeleton binding,” were also upregulated. Conversely, the biological processes associated with the regulation of “chemokine production” and the molecular function of “cytokine activity” were remarkably suppressed (Fig. 5D) (Table S9).

**Fig 6:**
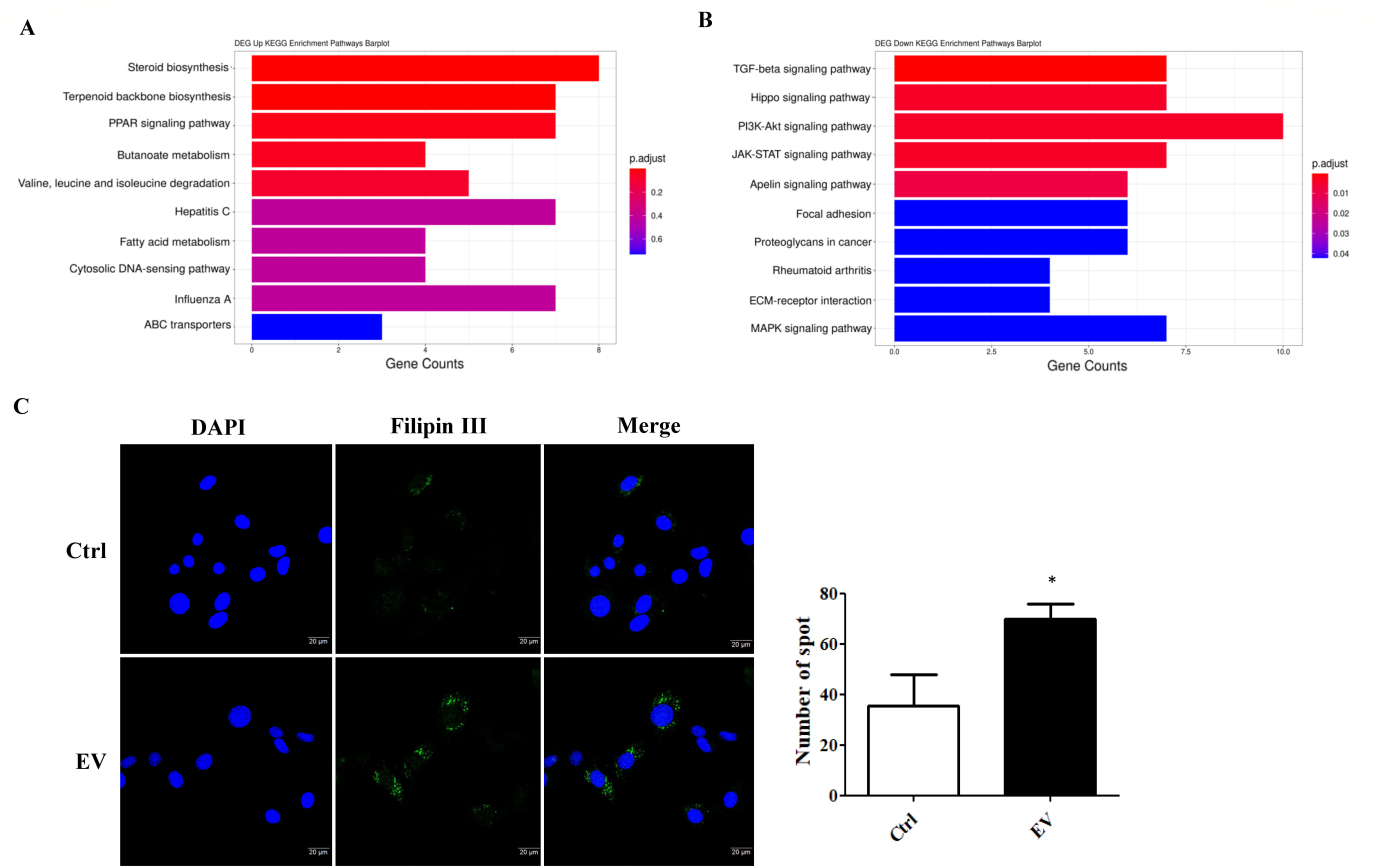
KEGG pathway enrichment analysis of mouse astrocytes treated with *A. cantonensis* L5 EVs reveals significant upregulation of cholesterol biosynthesis. (**A**) KEGG enrichment analysis revealed the upregulated pathways and (**B**) downregulated pathways in the transcriptomes of mouse astrocytes treated with *A. cantonensis* L5 EVs (60 µg/mL) for 12 h. (**C**) Mouse astrocytes were treated with *A. cantonensis* L5 EVs (60 µg/mL) for 12 h (EV), and cholesterol levels were determined by filipin III staining compared with the medium-treated control (Ctrl). Nuclei were stained with DAPI (blue). The right panel shows the quantitative results of punctate signals representing cholesterol levels in mouse astrocytes. Scale bar: 20 µm. *P < 0.05.

KEGG pathway mapping also showed that “steroid biosynthesis” was the most upregulated pathway (Fig. 6A), whereas the TGF-β, Hippo, PI3K-Akt, and JAK/STAT signaling pathways were most downregulated (Fig. 6B) (Tables S10 and S11). To functionally validate the RNA-seq data, we measured the abundance of cholesterol in L5 EV-treated cells compared with the medium-treated control. Filipin III interacts with the cholesterol molecules, exhibiting fluorescence upon binding to cholesterol (22). Notably, confocal images revealed that mouse astrocytes treated with L5 EVs significantly enhanced the punctate signals of filipin III compared with the medium-treated control (*P* < 0.05), suggesting that L5 EVs were able to induce cholesterol formation in the non-permissive hosts (Fig. 6C).

### *A. cantonensis* L5 EVs inhibit ATP-induced activation of the NLRP2 inflammasome in mouse astrocytes

**Fig 7:**
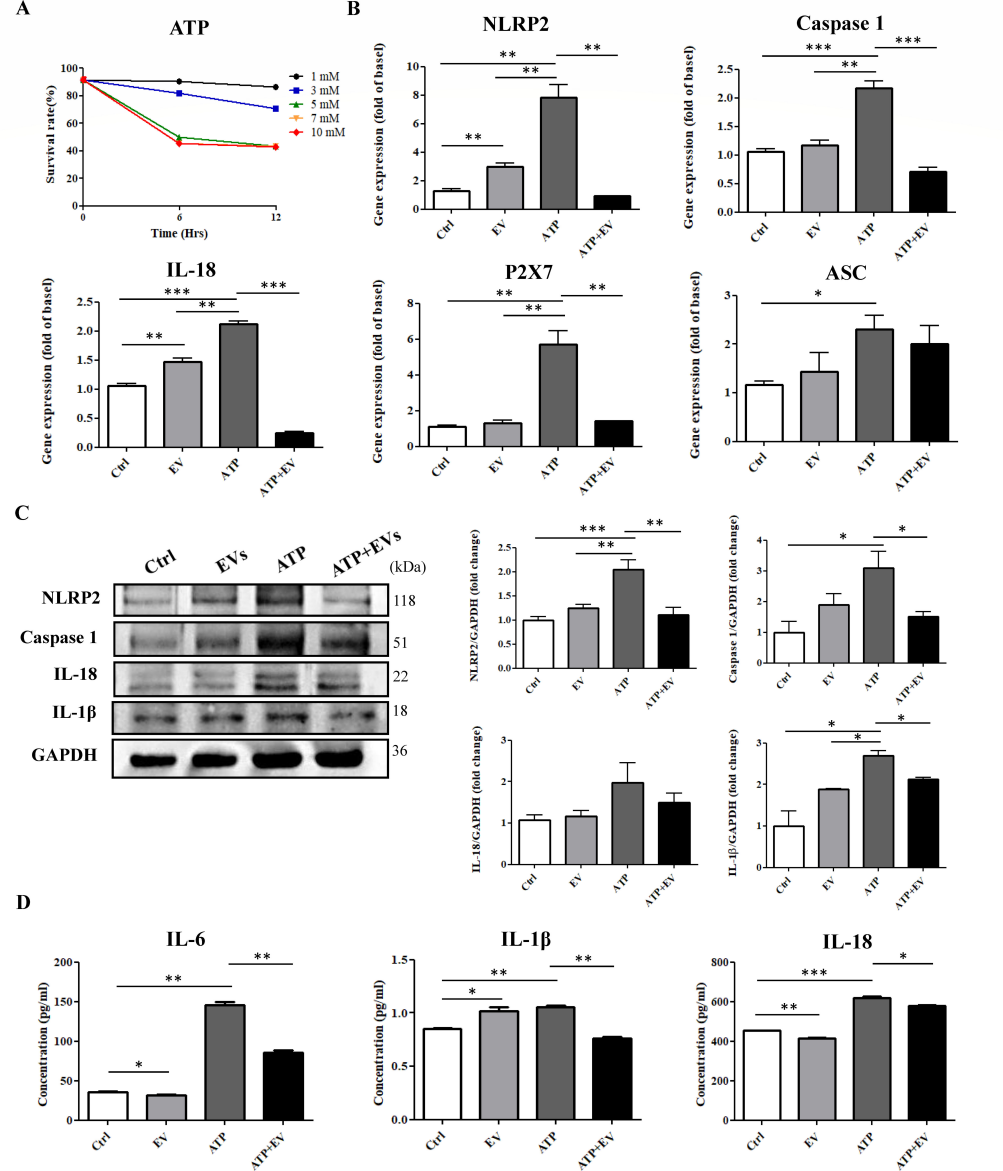
*A. cantonensis* L5 EVs alleviate ATP-induced NLRP2 inflammasome activation in mouse astrocytes. (**A**) The effects of different concentrations of ATP (1, 3, 5, 7, and 10 mM) on the survival of mouse astrocytes were determined by the CCK-8 assay. (**B**) To investigate the effects of L5 EVs on ATP-induced inflammasome activation, mouse astrocytes were pretreated with ATP (1 mM) for 1 h and then treated with L5 EV (60 µg/mL) (ATP + EV) for 12 h. The gene expression of NLRP2, capase-1, IL-18, P2X7, and ASC was determined in mouse astrocytes treated with L5 EVs (EV), ATP, and ATP + EV compared with the medium-treated control (Ctrl) by qPCR analysis. (**C**) The expression levels of NLRP2, capase-1, IL-18, and IL-1β proteins were determined in mouse astrocytes treated with EV, ATP, and ATP + EV compared with Ctrl by Western blot analysis. (**D**) The secretion of IL-6, IL-1β, and IL-18 was determined in mouse astrocytes treated with EV, ATP, and ATP + EV compared with Ctrl by ELISA. *P < 0.05 **P < 0.01, ***P < 0.001

As the gene expression of NLRP2 was downregulated in L5 EV-treated mouse astrocytes, we further investigated whether the NLRP2 inflammasome was suppressed in mouse astrocytes in response to L5 EVs treatment. The NLRP2 inflammasome activates caspase-1, leading to the processing and release of pro-inflammatory cytokines such as IL-1β and IL-18 (23). Additionally, the NLRP2 inflammasome represents an important branch of the glial innate immune inflammatory response in human astrocytes and can be activated by the danger associated molecular pattern (DAMP) ATP (24). Hence, we first examined the effects of different concentrations of ATP (1, 3, 5, 7, and 10 mM) on the viability of mouse astrocytes after 12 h of treatment (Fig. 7A). As mouse astrocytes treated with 1 mM ATP maintained viability above 80%, this concentration was used to investigate the activation of NLRP2 inflammasome-related molecules. Treatment of mouse astrocytes with *A. cantonensis* L5 EVs alone resulted in increased gene expression levels of NLRP2 and IL-18 (Fig. 7B), but did not alter the protein expression levels of NLRP2 inflammasome-related molecules, including NLRP2, caspase 1, IL-18, and IL-1β (Fig. 7C). Notably, ATP treatment upregulated both gene and protein expression levels of almost all NLRP2 inflammasome-related molecules (Fig. 7B and C), confirming that ATP serves as an effective inducer for the activation of the NLRP2 inflammasome in mouse astrocytes. It should be noted that the upregulation of ATP-induced NLRP2 inflammasome-related proteins, as well as the pro-inflammatory cytokines IL-6, IL-18, and IL-1β, was inhibited in mouse astrocytes treated with ATP in combination with *A. cantonensis* L5 EVs (Fig. 7C and D). Taken together, these results suggest that *A. cantonensis* L5 EVs potentially attenuate ATP-induced NLRP2 inflammasome activation and subsequent pro-inflammatory cytokine secretion in the non-permissive hosts.

## DISCUSSION

In the present study, we reported for the first time the isolation and characterization of *A. cantonensis* L5 and adult worm EVs. A previous study has examined the size and morphology of *A. cantonensis* L4 exosome (19), indicating that the exosomes are rounded structures with a size range of 60–200 nm, consistent with our findings for *A. cantonensis* L5 and adult worm EVs. Hence, most EVs isolated from different stages of *A. cantonensis* fall within the exosome size range. Interestingly, we found that the size of adult worm EVs is smaller than that of L5. Furthermore, we identified a great number of proteins in the EV cargo of L5 compared with that of adult worms. It has been reported that vesicle contents vary depending on the gender and lifecycle stage of the helminth. For instance, significant morphological diversity has been observed among EVs produced from eggs, juveniles, and adults of *F. hepatica* (25). Additionally, the EV proteome of the filarial nematode *B. malayi* is both stage- and sex-specific (26). These findings suggest that morphological diversity and varying contents of EVs derived from different lifecycle stages of the helminth may be attributed to various EV-producing cellular origins and the need to manipulate a changing host environment or interact with host cells (27, 28).

A previous proteomic analysis of *A. cantonensis* young adults identified disulfide isomerase and calreticulin as the most abundant proteins (13). Additionally, several proteins found in the ESPs of young adults also can be detected in the L5 EVs, suggesting that some ESPs may be packaged in EVs, while others are not. Our study found that Galectin (Gal) is highly enriched in L5 EVs, but not in adult EVs. This finding is consistent with a previous differential proteomic analysis that reported a higher Gal-1 expression level of *A. cantonensis* in L5 compared with L3 (29). *A. cantonensis* Gal-1 has been shown to induce apoptosis of macrophages by binding to annexin A2 (30). Upregulation of *A. cantonensis* Gal-1 has also been demonstrated to play a protective role against oxidative stress damage by reducing fat deposition, which may help *A. cantonensis* L5 survive immune attacks by eosinophils in the human central nervous system (20). Based on these findings, Gal packaged in L5 EVs is likely crucial for parasite virulence, immune regulation, or survival in the human host.

The transcriptomic data revealed that Na^+^/K^+^-transporting ATPase interacting 4 (NKAIN 4) is the most upregulated protein in mouse astrocytes in response to *A. cantonensis* L5-EV treatment. NKAIN 4 is a member of a family consisting of four mammalian proteins (NKAIN1, 2, 3, and 4), but the precise function of NKAIN remains unknown. NKAIN proteins have been shown to be neuronally expressed in multiple regions of the mouse brain, where they interact with the β1 subunit of the Na^+^/K^+^-ATPase (31). Na^+^/K^+^-ATPase is a membrane-bound enzyme essential for maintaining neuronal excitability. Exposure of rats to repeated restraint stress has been shown to reduce the levels of Na^+^/K^+^-ATPase in brain structures, leading to changes in both short- and long-term memory, learning, and exploratory response (32). Additionally, a decrease in brain Na^+^/K^+^-ATPase activity has been observed in experimental traumatic brain injury, accompanied by alterations in membrane fluidity and neuronal excitability is observed (33). Based on these findings, it is crucial to investigate the mechanism by which NKAIN 4 interacts with Na^+^/K^+^-ATPase, which will advance our understanding of the pathogenesis of brain injury caused by *A. cantonensis* L5-derived EVs.

We observed that 3-hydroxy-3-methylglutaryl-coenzyme A synthase 1 (HMGCS1) and HMGCS2 are upregulated in mouse astrocytes in response to *A. cantonensis* L5-EVs treatment (Table 3; Table S6). HMGCS is an enzyme that converts acetyl-CoA and acetoacetyl-CoA into HMG-CoA, an intermediate in both sterol synthesis and ketogenesis (34, 35). HMGCS1, located in cytoplasm, participates in sterol biosynthesis, whereas HMGCS2, expressed in the mitochondrial matrix, functions as a key enzyme for ketogenesis (36). Consistent with this finding, GO and KEGG functional analyses identify the sterol biosynthetic process as the most upregulated pathway in mouse astrocytes upon *A. cantonensis* L5-EV treatment, which has been validated by the enhanced formation of filipin III that binds to cholesterol. Similarly, a previous study demonstrated that EVs secreted by *B. malayi* microfilariae significantly upregulate the steroid biosynthetic process in the mosquito host (37). The production of host steroid hormones can be modulated by the development of parasites, leading to more rapid parasite development and longer-lasting infections (38). Therefore, it is likely that *A. cantonensis* L5-EVs overexpress the HMGCS-related genes to regulate steroid biosynthesis in mouse astrocytes, potentially playing an important role in the pathogenesis of *A. cantonensis*.

The expression levels of certain chemokines and their receptors are upregulated in *A. cantonensis* L5-EV-treated mouse astrocytes. For instance, chemokine (C-X-C motif) ligand 13 (CXCL13) is highly expressed in L5-EV-treated astrocytes (Table S6). CXCL13 is a key homeostatic chemokine constitutively expressed in lymphoid organs and also induced upon CNS inflammation, serving as a biomarker for Lyme neuroborreliosis (LNB), CNS lymphoma, and multiple sclerosis (MS) (39). Additionally, CXCL13 elevations in cerebrospinal fluid have been implicated in bacterial/viral and aseptic meningitis (40) and encephalitis (41). Hence, CXCL13 upregulation can also be considered as an inflammatory chemokine induced by *A. cantonensis* L5-EVs. Additionally, chemokine (C-X-C motif) receptor 1 (CXCR1) is also upregulated in L5-EV-treated astrocytes (Table S6). CXCR1 has been shown to play an crucial role in neuronal apoptosis induced by methamphetamine (METH) and may be a potential target for METH-induced neurotoxicity therapy (42). Furthermore, blockade of the CXCR1/2 receptors by reparixin, a noncompetitive allosteric antagonist of CXCR1 and CXCR2, promotes neuroprotective effects by reducing the levels of neutrophil infiltration in the brain and the tissue damage associated with middle cerebral artery occlusion and reperfusion (43). Hence, L5-EV-induced CXCL13 and CXCR1 may drive inflammation in mouse astrocytes.

We noted that potassium voltage-gated channel, subfamily F, member 1 (KCNF1) is significantly downregulated in L5-EV-treated mouse astrocytes. A previous RNA-seq analysis of DEGs within brain tissue during chronic stress-induced brain injury (CSBI) identified significant alterations in potassium channel function (44). Further functional assay demonstrated that retigabine, a voltage-gated potassium channel opener, has a protective effect in CSBI rats. Hence, it is of interest to investigate the role of KCNF1 in potassium homeostasis in mouse astrocytes in response to *A. cantonensis* L5-EV treatment, potentially clarifying the association of host potassium homeostasis with the pathogenesis of *A. cantonensis* infection.

Pathway enrichment analysis demonstrated that the TGF-β signaling pathway is the most downregulated pathway in mouse astrocytes treated with L5-EVs. The expression of TGF-β has been shown to be reduced in the hippocampus of stressed rats, concomitant with increased expression of inflammatory cytokines, such as IL-1β (45). Additionally, neurogenesis was found to be impaired, indicating a potential relationship between decreased TGF-β signaling and loss of neurogenic function. Another study indicated that neurons exposed to increased cortisol levels have decreased TGF-β expression and reduced neurogenesis (46). The neuroprotective effect of TGF-β1 has been shown to be associated with the inhibition of chemokines, including monocyte chemoattractant protein-1 (MCP-1) and macrophage inflammatory protein-1α (MIP-1α) during cerebral ischemia and reperfusion (47). As the modulation of TGF-β-signaling in traumatic brain injury (TBI) has both beneficial and detrimental effects (48), it remains to be determined whether the suppression of TGF-β-signaling by *A. cantonensis* L5 EVs is beneficial for the pathogenesis of parasite infection or represents a protective response by the host.

To date, limited data are available on the interaction of helminths with inflammasomes. It has been shown that a synthetic analog of E62, a glycoprotein secreted by the filarial nematode *Acanthocheilonema viteae*, suppresses arthritis in mice by preventing inflammasome activation (49). Additionally, *H. polygyrus* and their ESPs activate the NLRP3 inflammasome to elicit IL-1β secretion, which suppresses the release of IL-25 and IL-33, resulting in suboptimal type 2 immunity and allowing pathogen chronicity (50). Furthermore, the gastrointestinal helminth *Trichuris muris* secretes ESPs and exosomes that promote NLRP3-dependent IL-18 secretion, limiting both innate and adaptive T cell-mediated anti-parasitic immunity (51). A previous study reported that upon infection with the *A. cantonensis* L5, NLRP1B and NLRC4 are activated as the inflammasome activators, which in turn activate caspase-1 and the downstream secretion of inflammatory cytokines, including IL-1β and IL-18 (5). Our study unveiled for the first time that *A. cantonensis* L5 is able to suppress ATP-induced NLRP2 inflammasome activation in mouse astrocytes. Further investigation is necessary to determine whether this L5 EV-mediated reduction in inflammation in non-permissive hosts benefits the parasite by facilitating the establishment of chronic infection.

In conclusion, we successfully identified the protein compositions of EVs derived from L5 and adult worms. The distinct cargo in L5 and adult worm EVs implies different parasite pathogenicity and host responses. We verified the impact of L5 EVs on mouse astrocytes, demonstrating that L5 EVs enhance cholesterol synthesis and reduce ATP-induced NLRP2 inflammasome activation. These highly modulated host responses to L5 EVs likely play pivotal roles in the pathogenesis of *A. cantonensis* infection, warranting further investigation. Additionally, future studies will be needed to verify the specific roles of enriched proteins in L5 EVs, which may serve as significant virulence or immunomodulatory factors in the human host. These EV-containing molecules might contribute to the development of therapeutic targets or vaccine candidates against *A. cantonensis*.

## MATERIALS AND METHODS

### Animals

SD rats aged 6 weeks were purchased from BioLASCO Taiwan Co., Ltd. (Taipei, Taiwan). The rats were group-housed in plastic cages and provided with food and water *ad libitum*.

### Isolation and culture of *A. cantonensis* L5 and adult worms

A Taiwan strain of *A. cantonensis* was isolated from an *Achatina fulica* snail in Taipei and has been maintained in our laboratory since 1985. The third-stage larvae (L3) of *A. cantonensis* were isolated from infected snails by digestion with 0.6% pepsin (pH 2–3, Sigma-Aldrich, USA). Each SD rat was infected with 200 *A*. *cantonensis* L3 via stomach incubation using a feeding tube. Brain tissues were collected after anesthetizing with 3% isoflurane on day 21 post-infection. The living *A. cantonensis* L5 was collected from the brain tissues as previously described (12). On day 51 post-infection, adult worms of *A. cantonensis* were isolated from the lung and heart tissues. *A. cantonensis* L5 and adult worms were washed with normal saline and phosphate-buffered saline (PBS), transferred to Roswell Park Memorial Institute (RPMI) medium (Sigma-Aldrich, USA) containing 1% antimycotic solution (Sigma-Aldrich, USA)and cultured in a CO_2_ incubator at 37°C.

### Mouse astrocyte culture

A mouse brain astrocyte cell line (CRL-2535) was cultured in Dulbecco’s modified Eagle medium/nutrient mixture F-12 (DMEM/F-12) (Corning, USA), containing 10% fetal bovine serum (FBS) (Gibco, USA) and 10% antibiotic–antimycotic solution (Sigma-Aldrich, USA).

### Purification of *A. cantonensis* L5 and adult worm EVs

The culture supernatant of *A. cantonensis* L5 and adult worms was replaced every 2 to 3 days. The collected supernatant was filtered through a 0.22 µm filter and concentrated using centrifugal filter units (Amicon Ultra-15, Merk Millipore, USA). Approximately 2–3 mL concentrated supernatant was used for EV isolation with the Total Exosome Isolation Reagent (Invitrogen, USA). Briefly, the ratio of concentrated supernatant to reagent is 1:0.5. After incubation for 16 h at 4°C, the solution was centrifuged at 10,000 × *g* for 1 h. The pellet was collected and resuspended in 50–100 μL PBS. For the isolation of *A. cantonensis* L5 and adult worms EVs by ultracentrifugation, the concentrated secretory fraction was added to a polycarbonate bottle with cap assembly (Beckman Coulter) and centrifuged at 14,000 rpm for 2 h at 4°C using the Optima XL-100K Ultracentrifuge (Beckman Coulter).

### Transmission electron microscopy

*A. cantonensis* L5 and adult worm EVs were fixed with 2.5% glutaraldehyde and 3% formaldehyde for at least 6 h. The EV samples were then fixed with osmium tetroxide (OsO_4_) for 50–70 min and dehydrated with increasing concentrations of ethanol (50%, 75%, 85%, 95%, and 100%). The samples were embedded in Epon 812 and heated at 60°C for 48 h. Thick sections (200 µm) were obtained using an Ultracut with a diamond knife and stained with 0.5% toluidune blue for around 40 s to 1 min for initial observation under a microscope. Thin sections (80 µm) were then collected and placed on charged carbon-coated grids. The sections were stained with 1% uranyl acetate and examined using a transmission electron microscope (HITACHI HT-7700).

### Nanoparticle tracking analysis (NTA)

To analyze the concentration and particle size of *A. cantonensis* L5 and adult worm EVs, nanoparticle tracking analysis (NTA) was performed using the NanoSight NS300 instrument (NanoSight Ltd., Amesbury, UK).

### Internalization of *A. cantonensis* L5 EVs by mouse astrocytes

*A. cantonensis* L5 EVs were labeled using the PKH67 Green Fluorescent Cell Linker Kit (Sigma-Aldrich, USA) according to the manufacturer’s instructions with modifications. Briefly, L5 EVs were mixed with an equal volume of diluent C and PKH67 dye in 150 µL of PBS at room temperature for 3 min in the dark. The reaction was terminated by adding 0.1% bovine serum albumin (BSA). The PKH67-labeled L5 EVs were centrifuged at 14,000 rpm for 1 h at 4°C, and the pellets were washed with PBS at 14,000 rpm for 30 min at 4°C. The PKH67-labeled EVs were resuspended in PBS. Mouse astrocytes were treated with different concentrations of *A. cantonensis* L5 EVs (20 µg/mL, 60 µg/mL, and 100 µg/mL) for various time intervals (1, 6, and 12 h). Cellular uptake was visualized using a fluorescence microscope and a confocal microscope (ZEISS LSM780).

### Cell viability assay

Cell viability was measured by a colorimetric CCK-8 assay. Mouse astrocytes (1 × 10^7^ cells/mL) were seeded in 24-well plates overnight and treated with different concentrations of *A. cantonensis* L5 EVs (0, 20, 60, and 100 µg/mL) for various time intervals (6, 12, and 24 h). Subsequently, 20 μL of the CCK-8 reagent was added to each well, and absorbance was determined at 450 nm using a microplate reader after incubation for 2 h at 37°C.

### Proteomic analysis of *A. cantonensis* EVs

The dried peptide mixtures were reconstituted with 0.1% formic acid for analysis using a nanoLC–LTQ-Orbitrap Elite mass spectrometer (Thermo Fisher, San Jose, CA, USA) as previously described (37). The MS raw data files were processed with Proteome Discoverer Software (version 2.3, Thermo Fisher, San Jose, CA, USA) and searched against the UniProt database (extracted for *A. cantonensi*s, 14,504 sequences) using the Mascot search engine (Matrix Science, London, UK; version 2.5). Trypsin digestion allowed for one missed cleavage, with a peptide mass tolerance of 10 ppm and CID fragment ions tolerance set at 0.5 Da for peptide identification. The settings of fixed and variable modifications were carbamidomethyl and oxidized methionine/acetyl, respectively. Peptide-spectrum matches (PSMs) were then filtered based on high confidence and rank one peptide identification in the Mascot search to maintain an overall false discovery rate below 0.01. Proteins identified with a single peptide hit were excluded from the analysis.

### RNA extraction and RNA-sequencing

Total RNA was extracted using GENEzol TriRNA Pure Kit (Geneaid, Taiwan). Six RNA samples isolated from L5 EV- and non-treated astrocytes were analyzed by RNA-seq. RNA purity and quantification were assessed using SimpliNano - Biochrom Spectrophotometers (Biochrom, MA, USA). RNA degradation and integrity were monitored using Qsep 100 DNA/RNA Analyzer (BiOptic Inc., Taiwan). One microgram of total RNA was used as input material for the RNA sample preparations. Sequencing libraries were generated using the KAPA mRNA HyperPrep Kit (KAPA Biosystems, Roche, Basel, Switzerland) following the manufacturer’s recommendations, with index codes added to attribute sequences to each sample. Briefly, mRNA was purified from total RNA using magnetic oligo-dT beads. Captured mRNA was fragmented by incubation at high temperature in the presence of magnesium in KAPA Fragment, Prime, and Elute Buffer (1×). First-strand cDNA was synthesized using random hexamer priming. Combined second-strand synthesis and A-tailing converted the cDNA hybrid to double-stranded cDNA (ds-cDNA), incorporated dUTP into the second cDNA strand, and added dAMP to the 3′ ends of the resulting ds-cDNA. Double-stranded cDNA adapters with 3′ dTMP overhangs were ligated to the library insert fragments to generate the library fragments carrying the adapters. To select cDNA fragments of preferentially 300–400 bp in length, the library fragments were purified using the KAPA Pure Beads system (KAPA Biosystems, Roche, Basel, Switzerland). The library carrying appropriate adapter sequences at both ends was amplified using KAPA HiFi HotStart ReadyMix (KAPA Biosystems, Roche, Basel, Switzerland) along with library amplification primers. The strand marked with dUTP was not amplified, allowing for strand-specific sequencing. Finally, PCR products were purified using KAPA Pure Beads system and the library quality was assessed using the Qsep 100 DNA/RNA Analyzer (BiOptic Inc., Taiwan).

### Bioinformatics analysis

The raw data obtained from high-throughput sequencing using the Illumina NovaSeq 6000 platform were converted into raw sequenced reads via CASAVA base calling and stored in FASTQ format. Quality assessment of the FASTQ files was performed using FastQC and MultiQC (52). The raw paired-end reads were processed with Trimmomatic (v0.40) (53) to remove low-quality reads, trim adaptor sequences, and eliminate poor-quality bases. The resulting high-quality data (clean reads) was used for subsequent analysis. These read pairs from each sample were aligned to the *Mus musculus* reference genome (GRCm38) uisng HISAT2. Read counts for individual genes were obtained with FeatureCounts (54). For gene expression analysis, the “trimmed mean of M-values” normalization (TMM) was performed using DEGseq (55) without biological duplicate, while the “relative log expression” normalization (RLE) was performed using DESeq2 (56) with biological duplicate. DEGs between the two conditions were identified in R using DESeq2, which employs a model based on the negative binomial distribution and Poisson distribution (57–59). The resulting *P*-values were adjusted using the Benjamini and Hochberg method to control the false discovery rate (FDR). Gene Ontology (GO) and Kyoto Encyclopedia of Genes and Genomes (KEGG) pathway enrichment analysis of DEGs were conducted using the clusterProfiler package (60–62).

### NLRP2 inflammasome activator treatment in mouse astrocytes

Previous studies have demonstrated that human astrocytes express the NLRP2 inflammasome, which can be activated by the danger-associated molecular pattern (DAMP) ATP (24). The viability of mouse astrocytes exposed to various concentrations (1, 3, 5, 7, and 10 mM) of ATP (Invivogen, San Diego, CA) was assessed using the CCK-8 assay. Mouse astrocytes were cultured in six-well plates and stimulated with 1 mM ATP for 12 h h to activate the NLRP2 inflammasome. To investigate the impact of *A. cantonensis* L5 EVs on NRLP2 inflammasome activation, the expression levels of NLRP2, capase-1, IL-18, P2X7, and apoptosis-associated speck-like protein containing a caspase recruitment domain (ASC) genes were measured in mouse astrocytes pretreated with 1 mM ATP for 1 h and then treated with L5 EVs for 12 h compared with a medium-treated control using qPCR analysis.

### Quantitative real-time PCR (q-PCR)

Total RNA was extracted from mouse astrocytes treated with *A. cantonensis* L5 EVs using the GENEzol TriRNA Pure Kit (Geneaid, Taiwan). RNA concentration and quality were assessed using a spectrophotometer (NanoDrop 2000, Thermo Scientific). cDNA was synthesized with the iScript cDNA Synthesis Kit (Bio-Rad, USA). Real-time qPCR was performed using SYBR Green Supermix (Bio-Rad, USA) on the ABI PRISM 7000 Sequence Detection System (Applied Biosystems, CA, USA). GAPDH served as an internal control for normalization. The ΔCt method was used to quantify the relative abundance of target genes under each condition, and the expression changes were presented as fold change using the 2^-ΔCt^ method. Primer pairs are listed in Table S12.

### Western blot analysis

Protein samples were mixed with 5× sample buffer (125 mM Tris-HCl, 1% SDS, 20% glycerol, 0.05% bromophenol blue, 5% 2-mercaptoethanol) and heated at 95°C for 10 min. Whole cell lysates were separated on a 12% SDS-PAGE gel and transferred to a polyvinylidene difluoride (PVDF) membrane using the Mini Trans-Blot cell transfer unit (Bio-Rad, USA) under 200 mA for 35 min. The membranes were blocked with 5% BSA in TTBS buffer (Tris-buffered saline containing 0.1% Tween 20) for 1 h. The membranes were incubated with primary antibodies in blocking buffer at 4°C overnight. The primary antibodies used were rabbit anti-NLRP2 (1:1,000 dilution, Sigma-Aldrich, USA), rabbit anti-caspase-1 (1: 1,000 dilution, Abcam, UK), rabbit anti-IL-18 (1:2,000 dilution, Abcam, UK), rabbit anti-IL-1β (1:1,000 dilution, Abcam, UK), and mouse anti-GAPDH (1:10,000 dilution, Sigma-Aldrich, USA) antibodies. After being washed with TTBS for three times, the membranes were incubated with goat anti-rabbit IgG (1:10,000 dilution, Sigma-Aldrich, USA) or rabbit anti-mouse secondary IgG (1:10,000 dilution, Sigma-Aldrich, USA) secondary antibodies in blocking buffer for 1 h. The membranes were incubated with a 1:1 mixture of stable peroxide solution and enhanced solution (Millipore, USA). Protein bands were visualized using the Biospectrum Imaging System (UVP) and quantified with ImageJ software.

### Enzyme-linked immunosorbent assay (ELISA)

To investigate the effects of *A. cantonensis* L5 EVs on the production of cytokines (IL-6, IL-1β, and IL-18) in host cells, mouse astrocytes were seeded at a density of 2 × 10^5^ cells/well in 24-well plates and co-incubated with L5 EVs for 2, 4, 6, 8, and 12 h. After incubation, the supernatant was collected and stored at −20°C. The levels of IL-6, IL-1β, and IL-18 were measured using ELISA kits (R&D System, USA) and quantified by detecting absorbance at 450 nm with an ELISA reader.

### Cholesterol detection assay

Mouse astrocytes (3 × 10^4^ cells/well) were seeded in a chambered coverslip with eight wells (ibidi) and incubated at 37°C overnight. The cells were treated with 100 µL of L5 EVs (60 µg/mL) in serum-free medium and or PBS (control). After incubation for 12 h, filipin III stock solution, diluted 1:100 with cholesterol detection assay buffer, was added into the wells (100 µl/well) and incubated for 2 h in the dark. Filipin was used for its convenience in detecting unesterified cholesterol. After removing the culture medium, the cells were washed three times with cholesterol detection wash buffer. The signals of filipin III were immediately detected using a confocal microscope (Leica TCS SP8). Quantitation of cholesterol in mouse astrocytes was analyzed by using Imaris Cell Imaging software.

### Statistical analysis

Quantitative data are presented as the mean ± SD of three independent experiments unless otherwise indicated. Statistical significance between groups was evaluated using a two-tailed Student’s *t*-test. A *P*-value of less than 0.05 was considered statistically significant.

## Data Availability

Raw RNA-seq reads have been deposited in the Sequence Read Archive (SRA) under BioProject PRJNA1187695. The mass spectrometry proteomics data have been deposited to the ProteomeXchange Consortium via the PRIDE (63) partner repository with the data set identifier PXD057961.

## SUPPLEMENTAL MATERIAL

Supplemental Tables msystems.01014-24-s0001.xlsx Table S1 to S12.

ASM does not own the copyrights to Supplemental Material that may be linked to, or accessed through, an article. The authors have granted ASM a non-exclusive, world-wide license to publish the Supplemental Material files. Please contact the corresponding author directly for reuse.
